# IFN-γ Attenuates Eosinophilic Inflammation but Is Not Essential for Protection against RSV-Enhanced Asthmatic Comorbidity in Adult Mice

**DOI:** 10.3390/v14010147

**Published:** 2022-01-14

**Authors:** Abenaya Muralidharan, Md Bashir Uddin, Christopher Bauer, Wenzhe Wu, Xiaoyong Bao, Keer Sun

**Affiliations:** 1 Department of Pathology and Microbiology, University of Nebraska Medical Center, Omaha, NE 68198-5900, USA; abenaya.muralidharan@unmc.edu (A.M.); christopher.bauer@unmc.edu (C.B.); 2 Department of Microbiology and Immunology, University of Texas Medical Branch, Galveston, TX 77555-1017, USA; mduddin@utmb.edu; 3 Department of Pediatrics, The University of Texas Medical Branch, Galveston, TX 77555-0372, USA; wenwu@utmb.edu (W.W.); xibao@utmb.edu (X.B.)

**Keywords:** RSV, asthma, comorbidity

## Abstract

The susceptibility to respiratory syncytial virus (RSV) infection in early life has been associated with a deficient T-helper cell type 1 (Th1) response. Conversely, healthy adults generally do not exhibit severe illness from RSV infection. In the current study, we investigated whether Th1 cytokine IFN-γ is essential for protection against RSV and RSV-associated comorbidities in adult mice. We found that, distinct from influenza virus, prior RSV infection does not induce significant IFN-γ production and susceptibility to secondary *Streptococcus pneumoniae* infection in adult wild-type (WT) mice. In ovalbumin (OVA)-induced asthmatic mice, RSV super-infection increases airway neutrophil recruitment and inflammatory lung damage but has no significant effect on OVA-induced eosinophilia. Compared with WT controls, RSV infection of asthmatic *Ifng^−/−^* mice results in increased airway eosinophil accumulation. However, a comparable increase in eosinophilia was detected in house dust mite (HDM)-induced asthmatic *Ifng^−/−^* mice in the absence of RSV infection. Furthermore, neither WT nor *Ifng^−/−^* mice exhibit apparent eosinophil infiltration during RSV infection alone. Together, these findings indicate that, despite its critical role in limiting eosinophilic inflammation during asthma, IFN-γ is not essential for protection against RSV-induced exacerbation of asthmatic inflammation in adult mice.

## 1. Introduction

Asthma is the most common chronic disease in the world with more than 235 million people affected. In young children and immunocompromised patients, asthmatic disease is often complicated with common viral infections such as respiratory syncytial virus (RSV) [1,2,3,4,5]. While several studies have explored the causal role of a RSV infection in asthma development, wherein RSV infection occurs first, leading to lung development conducive for asthma, the mechanisms underlying the effect of RSV super-infection on augmentation of pre-existing asthma remains to be fully understood.

RSV infection occurs at a high rate among children under the age of 5 and can lead to decreased effectiveness of asthma medications such as steroids [6,7,8]. Conversely, adults normally do not exhibit severe illness from an RSV infection and experience milder exacerbation in asthmatic disease. Identifying the underlying protective mechanisms in adults that are absent in infants can provide an in-depth understanding of the mechanisms involved in this comorbidity. It has been suggested that a deficient T-helper cell type 1 (Th) cytokine IFN-γ production makes infants more susceptible to RSV infection and subsequent asthma development [9,10]. Thus, we wanted to determine whether competent IFN-γ signaling is essential for protection against RSV and asthma comorbidity in adult mice.

In addition to asthmatic comorbidity, RSV has been shown to increase host susceptibility to secondary bacterial infection [11]. Acute influenza A virus (IAV) infection is a significant cause of diseases even in adults. Furthermore, IAV-induced lung immune complications, such as secondary bacterial pneumonia, are more common causes of severe morbidity and mortality [12,13]. Using a mouse model of viral and bacterial coinfection, we and others have shown that IFN-γ production during IAV infection impairs innate defense against bacterial infection [14,15]. Thus, we were interested to know whether RSV infection in adult mice increases the production of IFN-IFN-γ and subsequent susceptibility to *Streptococcus pneumoniae* infection.

In the current study, we showed that, distinct from IAV, prior RSV infection does not induce significant IFN-γ production or impair acute bacterial clearance during secondary *S. pneumoniae* infection in adult mice. In both ovalbumin (OVA) or house dust mite (HDM)-induced asthmatic mice, we found that RSV infection induces neutrophil recruitment but has no significant impact on asthma-associated eosinophilia. Furthermore, our results indicated that, although IFN-γ inhibits eosinophilic inflammation, it is not essential for protection against RSV-induced asthma exacerbation in adult mice.

## 2. Materials and Methods

### 2.1. Murine Model of Viral and Bacterial Infection

Specific pathogen-free, age-matched male and female adult C57BL/6 WT, IFN-α/β receptor 1 gene-deficient (*Ifnar*^−/−^), and IFN-γ gene-deficient (*Ifng*^−/−^) mice were purchased from the Jackson Laboratory (Bar Harbor, ME) and bred at the University of Nebraska Medical Center and University of Texas Medical Branch (UTMB) following Animal Care and Use Committee (IACUC) guidelines. All animal experiments were approved by UNMC, and all experiments were carried out in accordance with UNMC and UTMB Assurance of Compliance with PHS Policy on Humane Care and Use of Laboratory Animals, which is on file with the Office of Protection from Research Risks, NIH.

Viral challenge was performed with 1 × 10^5^ PFU RSV-A2 (ATCC: VR-1540) or a sublethal dose of X31 (~5 × 10^3^ PFU/mouse) administered intranasally (i.n.) to anesthetized mice in 50 µL of sterile PBS. Of note, X31 is an H3N2 influenza A strain that only causes mild illness in mice [16].

RSV-A2 was grown in HEp-2 cells according to the supplier’s instructions and sucrose-purified for animal studies. HEp-2 (ATCC: CCL-23) cells were grown in Dulbecco’s Modified Eagle Medium (DMEM) supplemented with 1.5 g/sodium bicarbonate, 2 mM glutamine, 1 mM HEPES, 20 U/mL Penicillin, 0.02 mg/mL Streptomycin, and 10% FBS. 

To induce bacterial pneumonia, anesthetized mice were inoculated i.n. with 50 μL of PBS containing 1 × 10^4^ CFU of serotype 14 strain TJO983 [17]. Bacterial burdens in the bronchoalveolar Lavage Fluid (BALF) and lungs were measured by sacrificing infected mice at 24 h after infection and plating serial 10-fold dilutions of each sample onto blood agar plates [18].

### 2.2. Ovalbumin (OVA)-Induced Asthma

OVA immunizations were done with 0.1 mg/mL OVA adjuvanted with 5 mg/mL Imject^®^ Alum (Thermo Scientific) administered intraperitoneally in 200 µL of sterile saline at day 0 and day 7 [19]. Two weeks following the last immunization, mice were challenged with 1 mg/mL OVA administered i.n in 50 µL sterile PBS for 3 consecutive days while the mice were under isoflurane anesthesia. Three days after the last challenge, mice were challenged with 1 × 10^5^ PFU RSV-A2 administered intranasally in 50 µL of sterile PBS or PBS only (asthma-alone controls) while under ketamine-induced anesthesia and sacrificed 7 days post-infection. 

### 2.3. House Dust Mite (HDM)-Induced Asthma

HDM immunizations were done intranasally with equal proportions of *D. pteronyssinus* and *D. farinae* (Greer Laboratories Inc. (Lenoir, NC, USA)) diluted 4-fold in 100 µL sterile PBS administered once a week for 3 weeks while the mice were under isoflurane anesthesia [20,21]. Two weeks following the last immunization, mice were challenged with equal proportions of *D. pteronyssinus* and *D. farinae* diluted 4-fold in 100 µL sterile PBS for 2 consecutive days. Five days after the last challenge, mice were challenged with 1 × 10^5^ PFU RSV-A2 administered intranasally in 50 µL of sterile PBS or PBS only while under ketamine-induced anesthesia and sacrificed 7 days post-infection. 

### 2.4. Plaque Assay

Titers of RSV-A2 stocks and viral levels in the lungs of infected mice were determined by plaque assays on HEp-2 cell monolayers. Briefly, lungs were removed 7 days post RSV-A2 challenge for viral titration [22] and collected in PBS for mechanical homogenization. The homogenates were frozen at −80 °C until further use. Serial dilutions of the homogenates were done and incubated on HEp-2 cells for 2 h at 37 °C. A 1:1 overlay of 2× DMEM media and 0.8% agarose was added. Following 6 days of incubation, the overlay was removed and the cell monolayer was stained with crystal violet before counting plaques. Results are expressed as total PFU per mouse lung.

### 2.5. BALF Collection

BALF samples were collected by making an incision in the trachea and lavaging the lung twice with 0.8 mL PBS, pH 7.4. Following centrifugation, the cell pellet was used for flow cytometry analysis. Total leukocyte counts were determined using a hemacytometer.

### 2.6. Flow Cytometry

BALF cells were incubated with 2.4 G2 mAb against FcγRII/III [23] and stained with APC-conjugated anti-CD11c (Biolegend, San Diego, CA, USA), BUV395-conjugated anti-CD11b (BD Biosciences, Franklin Lakes, NJ, USA), FITC-conjugated or PE-Cy7-conjugated anti-Ly6G (clone 1A8, BD Biosciences, Franklin Lakes, NJ, USA), FITC-conjugated anti-MHCII (I-A/I-E), PerCP-Cy5.5-conjugated or PE-conjugated anti-Ly6C(Biolegend, San Diego, CA, USA), and BV421-conjugated (Biolegend, San Diego, CA, USA) or PE-conjugated anti-Siglec-F (BD Biosciences, Franklin Lakes, NJ, USA) mAbs. The stained cells were analyzed on BD LSRII-green using BD FACSDiva software. Data analysis was completed using FlowJo software.

### 2.7. Evaluation of Airway Damage

Total protein levels and lactic acid dehydrogenase (LDH) activities in BALF were analyzed by a Micro BCA protein assay kit (Thermo Scientific, Waltham, MA, USA) and a LDH cytotoxicity assay kit (Thermo Scientific, Waltham, MA, USA), respectively. 

### 2.8. Statistical Analysis

Significant differences between experimental groups were determined using an ANOVA analysis followed by Tukey’s multiple comparisons’ test in GraphPad Prism 6 software (La Jolla, CA, USA). For all analyses, a *p* value < 0.05 was considered to be significant.

## 3. Results

### 3.1. Prior RSV Infection Does not Have a Significant Impact on Lung Bacterial Clearance during Secondary Pneumococcal Infection

**Figure 1:**
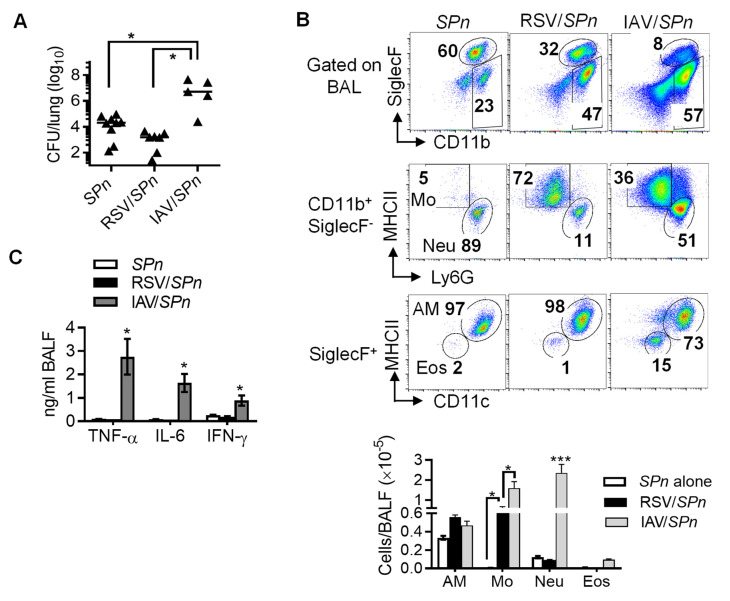
Prior RSV infection has no significant impact on acute bacterial clearance during secondary *S. pneumoniae* infection. C57BL/6 WT mice were infected with RSV or X31 (IAV) and 7 days later super-challenged with *SPn*. (**A**) Lung *SPn* burdens, (**B**) Representative dot plots (top panel) and numbers (bottom panel, mean ± SE, n ≥ 5) of airway alveolar macrophages (AM), monocytes (Mo), neutrophils (Neu), and eosinophils (Eos), and (**C**) levels of TNF-α, IL-6, and IFN-γ (mean ± SE, n ≥ 5) at 24 h after *SPn* infection. Each dot represents one mouse; * *p* < 0.05, *** *p* < 0.001, one-way ANOVA with Tukey’s multiple comparisons’ test. Data shown are representative of two independent experiments.

It has been well established that IAV infection impairs innate antibacterial immunity and thereby increases susceptibility to secondary bacterial pneumonia. We wanted to determine whether, like IAV, prior RSV infection impairs lung bacterial clearance during secondary *Streptococcus pneumoniae* infection. Accordingly, C57BL/6 WT mice were infected either with RSV-A2 or A/H2N3/X31 (IAV), and 7 days later super-challenged with *S. pneumoniae* serotype 14 strain TJO983 (*SPn*). As expected, prior IAV infection resulted in 10^3^-fold bacterial outgrowth in the lungs 24 h after *SPn* super-infection (Figure 1A). In contrast, RSV infection does not appear to affect acute bacterial clearance, as evidenced by comparable bacterial burdens in *SPn* single-infected and RSV/*SPn* coinfected mice.

Flow cytometry analysis of BALF cells revealed that prior RSV infection had no significant impact on the numbers of CD11c^+^Siglec-F^+^ alveolar macrophages (AM) and CD11b^+^Ly6G^+^ neutrophils (Neu) in response to *SPn* infection (Figure 1B). There were also limited numbers of airway CD11c^−^Siglec-F^+^ eosinophils in either *SPn* single-infected or RSV/*SPn* coinfected mice. However, the number of CD11b^+^Ly6G^−^MHCII^+^ monocytes (Mo) was increased in RSV/*SPn* coinfected mice as compared with *SPn* single-infected controls. The recruitment of inflammatory cells, particularly neutrophils, was significantly enhanced in response to IAV/*SPn* coinfection, in agreement with highly elevated inflammatory cytokine levels (Figure 1C). In contrast, RSV/*SPn* coinfected mice exhibited barely detectable IFN-γ, TNF-α, and IL-6 production, similar to *SPn* single-infected controls. Together, these results suggest that, compared with IAV, prior RSV infection has a very limited impact on the ability of bacterial control in adult mice.

### 3.2. RSV Infection Induces Neutrophil Recruitment and Increases Acute Lung Damage in OVA-Induced Asthmatic Mice 

**Figure 2:**
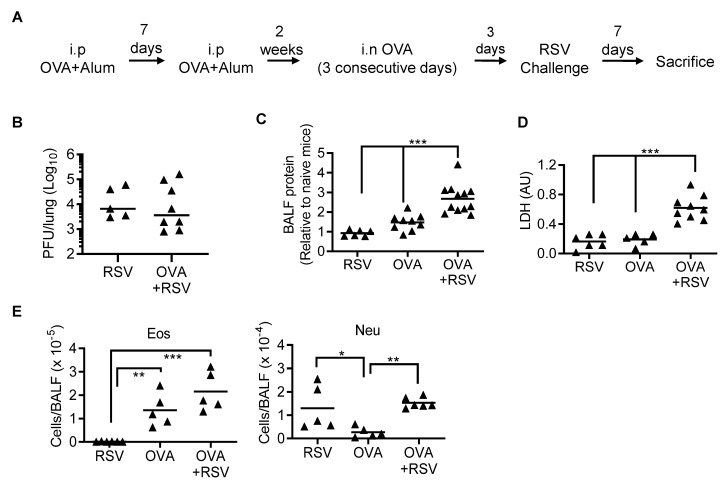
RSV infection of WT mice with OVA-induced asthma increases eosinophilic and neutrophilic inflammation. (**A**) Scheme of the experimental design. (**B**) Lung RSV titers, (**C**) airway protein and (**D**) LDH levels, (**E**) numbers of airway eosinophils (Eos) and neutrophils (Neu) in the BALF at day 7 after RSV infection of OVA-induced asthmatic C57BL/6 WT mice. Corresponding control mice were treated with PBS. AU, Arbitrary Unit; * *p* < 0.05, ** *p* < 0.01, *** *p* < 0.001, one-way ANOVA with Tukey’s multiple comparisons’ test. Data shown are representative of two independent experiments.

Chronic conditions such as asthma can be exacerbated to dangerous levels by viral infections. Particularly, RSV has been implicated in asthma disease exacerbation. We, thus, examined the effects of RSV super-infection in OVA-sensitized mice, the most common mouse model for studying asthma [24,25,26]. Specifically, after intraperitoneal (i.p.) OVA immunization, C57BL/6 WT mice were i.n. challenged with OVA to induce asthma, followed by RSV infection 3 days later (Figure 2A). We found that lung viral titers in day 7 RSV-infected asthmatic mice (OVA+RSV) were not significantly different from those of RSV-infected non-asthmatic (RSV alone) mice (Figure 2B). Conversely, RSV infection of asthmatic mice indeed resulted in increased airways’ protein and lactate dehydrogenase (LDH) levels (Figure 2C,D) compared with OVA asthma or RSV infection alone, suggesting that RSV enhances inflammatory lung damage in asthmatic mice.

We next examined the signature indicators of asthmatic inflammation, i.e., eosinophil and neutrophil recruitment, during RSV/asthma comorbidity. The respiratory lumen of naive mice contains predominantly AM and barely any eosinophils or neutrophils [15]. After immunization and challenge with OVA followed by a RSV challenge (OVA + RSV), WT mice exhibited increased inflammatory cells in the airways (Figure 2E). However, the numbers of eosinophils and neutrophils in OVA+RSV mice were comparable to OVA asthma and RSV infection-only controls, respectively. These results suggest that RSV super-infection of adult mice has no synergistic impact on the OVA-induced inflammatory cell recruitment, and vice versa.

### 3.3. IFN-γ Inhibits Eosinophilic and Neutrophilic Inflammation during RSV Infection of OVA-Induced Asthmatic Mice

**Figure 3:**
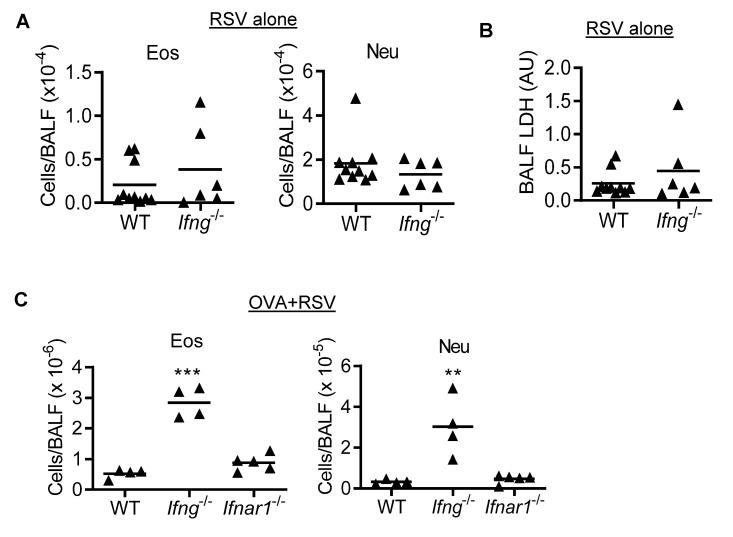
IFN-γ inhibits both eosinophilic and neutrophilic inflammation during RSV infection of OVA-induced asthmatic mice. (**A**) Numbers of eosinophils (Eos) and neutrophils (Neu) and (**B**) LDH levels in the BALF at day 7 after RSV infection of C57BL/6 WT and *Ifng*^−/−^ mice. (**C**) Numbers of airway eosinophils and neutrophils at day 7 after RSV infection of OVA-induced asthmatic C57BL/6 WT, *Ifng*^−/−,^ and *Ifnar*^−/−^ mice. ** *p* < 0.01, *** *p* < 0.001, one-way ANOVA with Tukey’s multiple comparisons’ test. Data shown are representative of two independent experiments.

To determine whether IFN-γ is responsible for protection against the synergistic impact of asthma and RSV on inflammatory cell recruitment in adult WT mice, we next investigated asthma and RSV-induced eosinophil and neutrophil infiltration in *Ifng*^−/−^ mice. Based on the hypothesis, we expected to detect a synergistic impact of RSV on asthmatic inflammation in the absence of IFN-γ. The numbers of airway eosinophils and neutrophils, as well as LDH levels, were comparable between *Ifng*^−/−^ and WT mice during RSV infection alone (Figure 3A,B). In addition, no significant differences in lung viral titer were observed between WT and *Ifng*^−/−^ mice (data not shown), suggesting a limited role of IFN-γ during RSV infection alone. Interestingly, both eosinophils and neutrophils were significantly increased during RSV infection of OVA-induced asthmatic *Ifng*^−/−^ animals, as compared with corresponding *Ifnar1*^−/−^ and WT mice (Figure 3C). Together, these results suggest that IFN-γ inhibits inflammatory cell recruitment during asthma and RSV comorbidity.

### 3.4. IFN-γ Inhibits Eosinophil Recruitment during Asthmatic Inflammation in the Absence of RSV Infection

**Figure 4:**
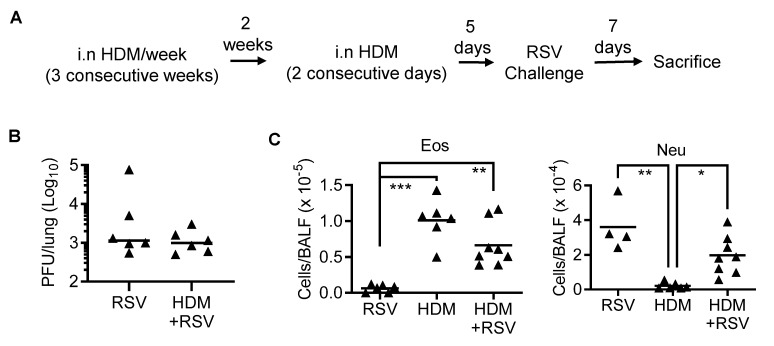
RSV infection of WT mice with HDM-induced asthma induces moderate levels of eosinophilic and neutrophilic inflammation. (**A**) Scheme of the experimental design. (**B**) Lung RSV titers and (**C**) numbers of airway eosinophils and neutrophils at day 7 after 10^5^ PFU RSV infection of HDM-induced asthmatic C57BL/6 WT mice. * *p* < 0.05, ** *p* < 0.01, *** *p* < 0.001, one-way ANOVA with Tukey’s multiple comparisons’ test. Data shown are representative of at least two independent experiments.

The HDM asthma model is considered a more relevant mouse model compared to OVA as it shares many similarities to human allergic asthma [24]. We, thus, examined the impact of RSV on inflammatory cell infiltration in HDM-induced asthmatic mice to further clarify the role of IFN-γ in asthma and RSV comorbidity (Figure 4A). Similar to observations in OVA-induced asthma model, HDM pre-condition had no significant impact on RSV clearance in the lung (Figure 4B). Conversely, a lower level of eosinophilia was detected in HDM immunized and challenged mice compared to OVA-induced asthmatic animals (Figure 4C). Nonetheless, in line with the observations in the OVA model, RSV infection does not appear to affect HDM-induced eosinophil recruitment, nor does HDM-induced asthma have a significant impact on RSV-induced neutrophil recruitment into the airways.

**Figure 5:**
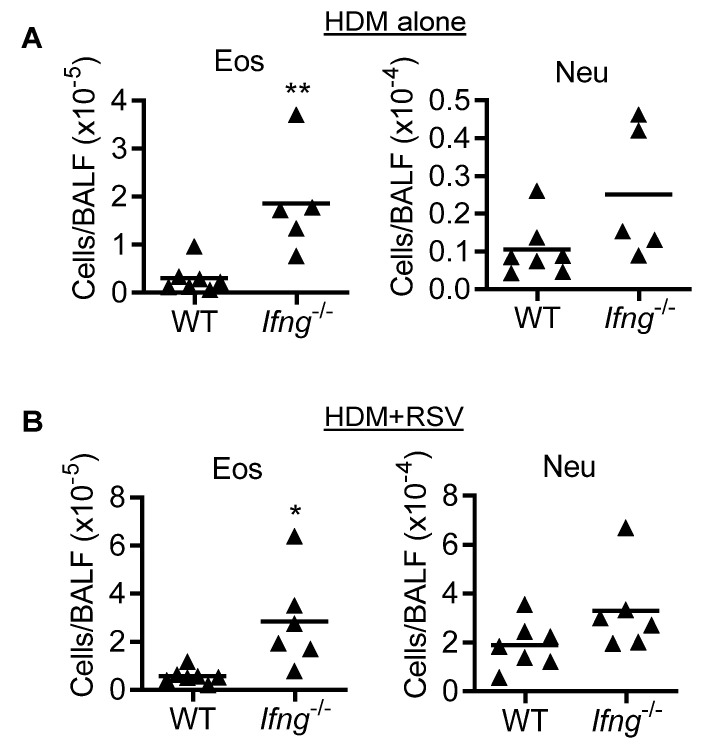
IFN-γ is necessary for limiting HDM-induced eosinophil infiltration regardless of RSV infection. Numbers of airway eosinophils and neutrophils in C57BL/6 WT and *Ifng^−/−^* mice during (**A**) HDM-induced asthma alone and (**B**) RSV infection of HDM-induced asthmatic mice. * *p* < 0.05, ** *p* < 0.01, one-way ANOVA with Tukey’s multiple comparisons’ test. Data shown are representative of at least two independent experiments.

Compared with HDM-induced asthmatic WT controls, HDM immunization and challenge of *Ifng*^−/−^ mice resulted in ~6-fold increased eosinophil infiltration (Figure 5A). These observations, together with findings in OVA asthma model, suggest that IFN-γ inhibits eosinophilic inflammation during asthma. Of note, during RSV infection of HDM-induced asthmatic (HDM + RSV) mice, we detected a comparable IFN-γ-mediated inhibition of eosinophilia, as evidenced by ~5-fold increases of airway eosinophils in *Ifng*^−/−^ mice as compared with HDM+RSV-treated WT controls (Figure 5B). Conversely, distinct from findings in OVA-induced asthmatic mice, there was no significant increase in neutrophils in the HDM+RSV group of *Ifng*^−/−^ mice as compared to WT animals (Figure 5B). Together, these results suggest that IFN-γ is dispensable for preventing the synergy of asthma and RSV on inflammatory cell recruitment in adult mice, despite its critical role in inhibiting asthma-induced eosinophilic inflammation.

## 4. Discussion

In the current study, we showed that, in contrast to IAV, prior RSV infection does not induce apparent susceptibility to secondary pneumococcal pneumonia in adult mice. On the other hand, in both OVA- and HDM-induced asthmatic mouse models, we found that RSV infection induces neutrophil recruitment but has no significant impact on asthma-induced eosinophilia, and vice versa. These findings suggest that RSV infection does not have a synergistic impact on asthmatic inflammation in adult mice. Further studies indicate that, while IFN-γ plays a critical role in the inhibition of airway eosinophil infiltration during asthma, it is not required for protection against asthma and RSV comorbidity in adult mice.

Chronic conditions such as asthma can be exacerbated to dangerous levels by viral infections. Indeed, RSV has been implicated with asthma at early age in two ways: first, in asthma development and, second, in asthma disease exacerbation. While the causal role of an RSV infection in asthma development has been well characterized, the mechanisms underlying the effect of RSV super-infection on the augmentation of pre-existing asthma remain to be fully clarified.

A deficiency in IFN-γ response has been associated with debilitating health of RSV-infected infants [27]. As such, a lower IFN-γ response in blood mononuclear cells of infants hospitalized with RSV infection has been correlated with increased disease severity [27,28]. Moreover, the numbers of IFN-γ producing γδ T cells were lower in infants hospitalized with RSV bronchiolitis compared to normal infants, which was accompanied by increased Th2 cytokine levels and recurrent wheezing in more than half the cohort [29]. Considering that RSV does not cause serious illness or exacerbation of asthma in healthy adults [7,30], we were interested to know whether competent IFN-γ signaling is responsible for protection against RSV and asthma comorbidity in adult mice.

Mouse models have offered important insights into the pathophysiology of asthma. There are two commonly used allergic asthma models, induced by OVA or HDM. Although OVA sensitization is more commonly used and classic inflammatory signs of asthma are effectively induced, the need for an alum adjuvant and the intraperitoneal route of immunization renders this model less translatable to human diseases. On the other hand, HDM extract, especially from *D. pteronyssinus* and *D. farinae*, has been identified as the most important indoor allergen in humans [31,32,33]. HDM is also administered intranasally, which is a more relevant route of administration for allergic asthma.

Accordingly, in the current study, we examined the impact of RSV infection on pre-existing asthma in both OVA and HDM models to determine whether competent IFN-γ response is essential for protection against asthma and RSV comorbidity in adults. We found that both OVA- and HDM-sensitized mice exhibited eosinophilic inflammation in the airway. By using *Ifng*^−/−^ adult mice, we attempted to mimic the low IFN-γ environment in infants with pre-existing asthma, which may lead to augmented eosinophilia following RSV infection. Indeed, we detected a significant increase in both eosinophils and neutrophils in OVA+RSV challenged *Ifng*^−/−^ mice, as compared with *Ifnar^−/−^* or WT controls, indicating an IFN-γ-specific inhibition of asthma exacerbation (Figure 3).

In the HDM-induced asthma and RSV comorbidity model, we only detected increased eosinophils due to IFN-γ gene-deletion. However, HDM-induced asthmatic *Ifng^−/−^* mice exhibited a comparable increase in eosinophilia even in the absence of RSV infection, suggesting an inhibitory effect of IFN-γ at the asthmatic phase. Furthermore, we did not observe any differences in lung viral titers between RSV infection alone and asthma/RSV comorbidity models in WT or *Ifng*^−/−^ mice. Additionally, no changes in lymphocyte population, serum IgG, and IgE levels were observed in asthma alone and OVA+RSV comorbidity model in WT and *Ifng*^−/−^ mice (data not shown). We also did not observe any changes in OVA- or HDM-elicited cytokines following RSV infection, as seen in other studies [6]. Together, these observations suggest that IFN-γ is dispensable for protection against RSV-associated morbidities in adult mice.

In addition, we previously showed that IAV-induced IFN-γ impairs innate antibacterial immunity against pneumococcal infection. Using a similar coinfection model, we found that prior RSV infection does not induce significant IFN-γ production and apparent suppression of lung bacterial clearance during secondary pneumococcal infection. The limitation of our coinfection model is that, compared to BALB/c mice, C57BL/6 mice used in this study are relatively more resistant to post-RSV pneumococcal infection [34]. Furthermore, it has been shown that RSV infection indeed delays bacterial clearance, when adult mice were super-infected with a high dose (5 × 10^7^ CFU/mouse) of *S. pneumoniae* [11]. Nonetheless, bacterial super-infection does not appear to have a significant impact on lung RSV clearance under these different infectious conditions [11], similar to our findings in IAV/*S. pneumoniae* coinfection model [18].

Using individual gene-deficient mouse models, we showed that IFN-γ and IFN-α/β receptor signaling is dispensable for protection against RSV-associated morbidities in adult mice. However, two IFN pathways may play compensatory protective roles in adult mice. It is also possible that other protective mechanisms, such as T regulatory cells, are responsible for suppression of RSV-associated morbidities, only in the absence of IFN-γ. Importantly, the current findings in adult mice do not exclude the possibility that IFN-γ is essential for protection against RSV/asthma comorbidity at early age.

In conclusion, the current study sheds light on the role of IFN-γ in preventing asthma and RSV/asthma comorbidity. Our findings indicate that, despite its critical role in limiting eosinophilic inflammation during asthma, IFN-γ is not essential for protection against RSV-induced exacerbation of asthmatic inflammation in adult mice.

## Data Availability

Not applicable.
